# Japanese population norms for preference-based measures: EQ-5D-3L, EQ-5D-5L, and SF-6D

**DOI:** 10.1007/s11136-015-1108-2

**Published:** 2015-08-25

**Authors:** Takeru Shiroiwa, Takashi Fukuda, Shunya Ikeda, Ataru Igarashi, Shinichi Noto, Shinya Saito, Kojiro Shimozuma

**Affiliations:** Department of Health and Welfare Services, National Institute of Public Health, 2-3-6 Minami, Wako, Saitama 351-0197 Japan; School of Pharmacy, International University of Health and Welfare, Otawara, Japan; Graduate School of Pharmaceutical Sciences, The University of Tokyo, Tokyo, Japan; Department of Health Sciences, Niigata University of Health and Welfare, Niigata, Japan; Graduate School of Health Sciences, Okayama University, Okayama, Japan; Department of Biomedical Sciences, College of Life Sciences, Ritsumeikan University, Kyoto, Japan

**Keywords:** EQ-5D, SF-6D, Health-related quality of life, Population norms, QALY, Japan

## Abstract

**Purpose:**

The purpose of this study was to measure the population norms for the Japanese versions of preference-based measures (EQ-5D-3L, EQ-5D-5L, and SF-6D). We also considered the relations between QOL score in the general population and socio-demographic factors.

**Methods:**

A total of 1143 adult respondents (aged ≥ 20 years) were randomly sampled from across Japan using data from the Basic Resident Register. The health status of each respondent was measured using the EQ-5D-3L, EQ-5D-5L, and SF-6D, and responses regarding socio-demographic data as well as subjective diseases and symptoms were obtained. The responses were converted to a QOL score using Japanese value sets.

**Results:**

The percentages of respondents with full health scores were 68 % (EQ-5D-3L), 55 % (EQ-5D-5L), and 4 % (SF-6D). The QOL score measured using the SF-6D was significantly lower than those measured using either EQ-5D score. The QOL score was significantly lower among respondents over the age of 60 years, those who had a lower income, and those who had a shorter period of education. Intraclass correlation coefficient showed a poor agreement between the EQ-5D and SF-6D scores. The differences in QOL scores between respondents with and those without any disease were 0.064 for the EQ-5D-3L, 0.061 for the EQ-5D-5L, and 0.073 for the SF-6D; these differences are regarded as between-group minimal important differences in the general population.

**Conclusion:**

The Japanese population norms of three preference-based QOL measures were examined for the first time. Such information is useful for economic evaluations and research examining QOL score.

## Introduction

When economic evaluations of healthcare technologies are performed, the incremental cost-effectiveness ratio (ICER) is regarded as a standard calculation. Various outcomes can be used as the denominator of ICER, but quality-adjusted life year (QALY) is widely applied for various areas of cost-effectiveness analysis. One reason is that quality of life (QOL) is one of the most important outcomes for not only medical interventions, but also healthcare policies. To calculate the QALY, the QOL score must be measured on a scale of 0 (death) to 1 (full health). Preference-based measures, such as the EuroQol 5-dimension (EQ-5D) [1, 2], the Health Utilities Index (HUI) [3–5], and the Short Form 6-dimension (SF-6D) [6–8], have been developed to calculate QOL scores. These measures were originally developed in English but have been translated into many languages. Japanese value sets for the EQ-5D (3L [9] and 5L [10]) and the SF-6D [11] have also been developed.

The mean QOL score in the general population is normally <1 because some people will have a less than full health score. People with diseases or symptoms are likely to continue living in their local community. Others may not report their health state as full health even if they do not have any diseases. Such reductions in QOL should be reflected in QALY calculations for economic evaluations. In addition, to interpret QOL scores obtained through a survey, it is important to be compared with the score for the general population as a reference value. Therefore, the *population norms*, which have been previously defined as “population reference data… for a specific country or international region” [12], used for preference-based measures are essential for both researchers and policymakers. The norms for these measures, especially for the EQ-5D-3L, have already been reported in many countries, including the UK [13], USA [14, 15], six European countries (Belgium, France, Germany, Italy, Netherlands and Spain) [16, 17], Spain (Catalonia) [18], Switzerland (French-speaking population) [19], Finland [20], Denmark [21], Portugal [22], Poland [23], Canada (Alberta) [24], Australia (Queensland) [25], China [26], Taiwan [27], Singapore [28, 29], Sri Lanka [30], and Brazil [31]. The population norms for the SF-6D have also been investigated in some countries, including the UK [32], USA [15], Australia [33], Portugal [34], and Brazil [35]. However, the Japanese population norms for QOL scores do not currently exist, with the exception of surveys performed in three areas [12] that were originally performed to obtain a value set [9]. Few standard norms for the EQ-5D-5L, a newly developed measure by the EuroQol Group, have been reported across the world.

The population in Japan was about 12.5 million in 2015, and almost all of the population speaks Japanese. Therefore, Japanese versions of the EQ-5D-3L, EQ-5D-5L, and SF-6D are widely used for calculating QOL scores in Japan, and Japan’s economic evaluation guideline [36] recommends the use of measures with value sets developed in Japan. The Ministry of Health, Labour and Welfare (MHLW) of Japan has collected data on these measures based on our concept. They also collected responses to a questionnaire included in the National Livelihood Survey, which Japan’s MHLW performs annually. This questionnaire includes questions regarding disease types and subjective symptoms.

Therefore, the objective of this study was to analyze data to obtain the population norms for the Japanese versions of three preference-based measures: the EQ-5D-3L, EQ-5D-5L, and SF-6D. The second objective was to examine the characteristics of each measure and the relations among measures. We also aimed to present the relation between the QOL score for the general population and characteristics such as sex, age, diseases, symptoms, and other socio-demographic factors.

## Methods

### Sampling

Data in this study came from MHLW’s survey, which took a representative sample. In the survey, a total of 1000 adult respondents (aged ≥ 20 years) were targeted in a random sampling from 100 sites (municipalities). The method used to select the 100 sites was as follows: First, the number of sites in each region (8 regions) was determined in proportion to the population of each region. Then, in every region, the number of sites belonging to each stratum (prefecture × size of municipalities) was calculated based on the populations of the stratum. The surveyed district (Cho-me, in Japanese) was randomly determined in a manner corresponding to the allocated number of sites in each stratum. Respondents were also randomly sampled from each selected district, stratified according to sex and age. People in a hospital or a nursing home were not included.

The Basic Resident Register can be used to select respondents living on each street in a random manner. In Japan, each municipality has its own Basic Resident Register data, which includes information on the name, sex, address, and date of birth of all residents. Each municipality has permitted the use of such data for public surveys. A door-to-door survey was performed from January to March in 2013. Investigators visited the registered addresses and distributed the questionnaire. They then collected the questionnaires a few days later and checked for any apparent errors (placement method). These visits continued until the planned number of responses was collected for each district. The investigators obtained the informed consent of all the respondents.

### Measures

Health status was measured using the EQ-5D-3L, EQ-5D-5L, and SF-6D. The respondents were presented with the EQ-5D-5L, EQ-5D-3L, and SF-6D (SF-36) in a fixed order. In addition, socio-demographic data for the respondents, such as sex, age, education, marital status, employment, and household income, were also collected.

The EQ-5D was developed by the EuroQol Group. The original version of the EQ-5D (now called the EQ-5D-3L) is comprised of five items: “mobility,” “self-care,” “usual activities,” “pain/discomfort,” and “anxiety/depression” assessed at three levels of description. To improve the lack of a sufficient sensitivity and the ceiling effect of the EQ-5D-3L, the newly developed EQ-5D-5L [37] has increased the number of levels for each health dimension from three to five.

The SF-6D is a measure for converting responses to the SF-36 (or SF-12 [38]) to a preference-based QOL score for economic evaluation. The SF-36 [39–41] is the most widely used measure for assessing health states in the world. Responses to selected items of the SF-36 can be classified according to descriptions of the SF-6D system, which consists of six dimensions [physical functioning (PF), role limitation (RL), social functioning (SF), bodily pain (BP), mental health (MH), and vitality (VT)] with five or six levels (defining a total of 22,500 health states). As the direct use of the SF-6D questionnaire is not recommended, we used the Japanese SF-36, version 2 [42].

The questionnaire also included a part of the National Livelihood Survey, which Japan’s MHLW performs annually. The questionnaire asks respondents whether they have any diseases for which they consult a doctor or not and whether they have any subjective symptoms or not. If they answer “yes,” they must then select the most important diseases and symptoms that they exhibit from a list of forty symptoms (having a fever, feeling sluggish, sleeplessness, etc.) and diseases (diabetes, obesity, hyperlipidemia, etc.).

### Statistical analysis

The responses obtained for the EQ-5D-3L, EQ-5D-5L, and SF-6D were first converted to QOL scores based on the Japanese value sets. Summary statistics for the QOL scores were calculated according to sex and age category (20–29, 30–39, 40–49, 50–59, 60–69, and 70 years and older). The percentage of people reporting any problem in each dimension was calculated after stratifying the subjects according to sex and age category. Chi-square tests (or the Fisher exact test if the expected frequency was low) were applied to determine the significance between the frequency of respondents with any problem and sex or age. The McNemar test was performed to confirm the frequencies of respondents with any problem in the EQ-5D-3L and the EQ-5D-5L. The intraclass correlation coefficient (ICC) was used for reliability between the three measures in addition to the Bland–Altman plot [43]. In the Bland–Altman plot, the average of the two measures was plotted on the x-axis, and the difference between the two measurements on the y-axis was used to check for systematic errors.

To detect the influence of socio-demographic factors and diseases/symptoms on the QOL scores, these variables were added (in addition to sex and age) to an analysis of variance (ANOVA). Diseases and symptoms for which more than 10 respondents had responded positively or that had a significant influence on the QOL score were included in the above statistical model. The influence of each disease and symptom was estimated using an ANOVA that included all the pertinent variables. The significance level was set at 0.05. Statistical analyses were performed using SAS 9.4.

We compared the QOL scores of the respondents between those with any subjective diseases/symptoms and those without using an ANOVA model. The difference was interpreted as the between-group minimal important difference (MID) of each preference-based measure in the general population. The MID, which corresponds to the smallest improvement considered to be worthwhile by a patient, is normally measured using a distribution-based or anchor-based method. Reportedly, “anchor-based differences can be determined either cross-sectionally at a single time point or longitudinally across multiple time points” [44]. The former cross-sectional anchor-based method was applied to our data, as the diseases and subjective symptoms were regarded as the anchors for the between-group MID.

This analysis was approved by the Ethics Committee of the National Institute of Public Health.

## Results

### Socio-demographic factors

Table 1 shows the socio-demographic factors of the sampled respondents. In total, the responses of 1143 respondents were randomly collected. In 2013, 4.3 % of the Japanese population lived in Hokkaido region, 7.1 % lived in Tohoku, 33.5 % lived in Kanto, 16.9 % lived in Chubu, 17.8 % lived in Kinki, 5.9 % lived in Chugoku, 3.1 % lived in Shikoku, and 11.4 % lived in Kyushu. The actual Japanese median household income was JPY 4.3 million, while the average was JPY 5.4 million in 2012. Married and unmarried people accounted for 61.1 and 22.8 % of the population, respectively. Overall, 19.1 % had graduated from university. Note that this statistic reflects the actual distribution of the population, but we sampled the same number of respondents from each age category. This means that the percentage among younger people was higher than that of the entire Japanese population. Based on the responses to the National Livelihood Survey, 48.2 % of the respondents had some disease for which they were consulting a doctor, while 48.6 % had some symptoms.

**Table 1:** Socio-demographic characteristics of respondents

	*N*	%
Age		
20–29	198	17.3
30–39	162	14.2
40–49	183	16.0
50–59	190	16.6
60–69	202	17.7
≥70	208	18.2
Sex		
Male	558	48.8
Female	585	51.2
Region		
Hokkaido	40	3.5
Tohoku	84	7.4
Kanto	383	33.5
Chubu	193	16.9
Kinki	205	17.9
Chugoku	64	5.6
Shikoku	34	3.0
Kyushu	140	12.3
Household income (JPY 10,000)		
<100	42	4.2
100–200	84	8.5
200–400	245	24.7
400–600	232	23.4
600–1000	257	25.9
1000–1500	101	10.2
1500–2000	18	1.8
>2000	13	1.3
Employment		
Full-time worker	429	37.8
Part-time worker	164	14.4
Self-employed or manager	114	10.0
Housemaker	231	20.3
Retired	126	11.1
Others	27	2.4
Education		
Elementary or junior high school	128	11.3
High school	468	41.2
College	237	20.9
University or graduate	291	25.6
Current student	11	1.0
Marital status		
Married	753	66.1
Unmarried	251	22.0
Divorced/bereaved	136	11.9

### QOL score and relation to socio-demographic factors

**Fig. 1:**
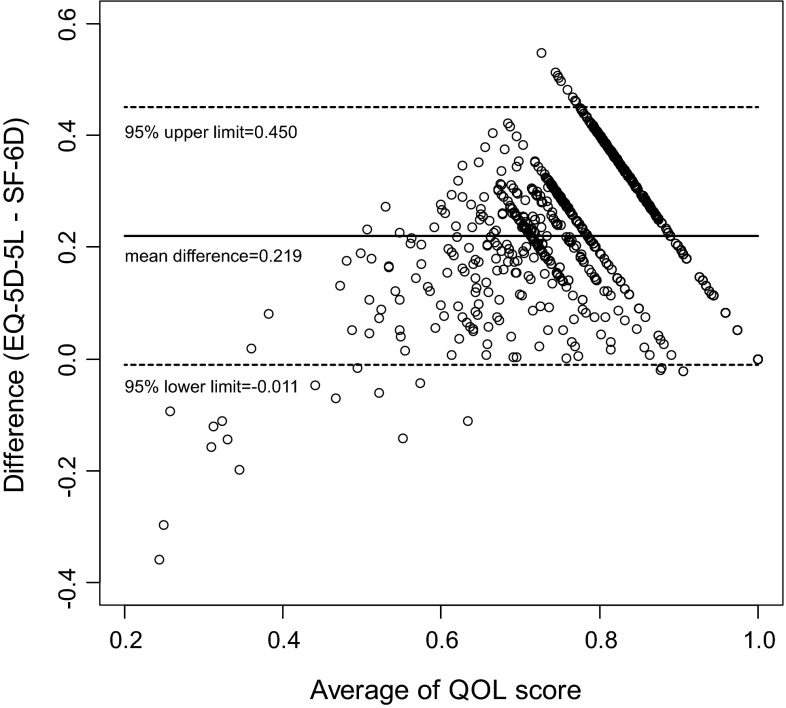
Bland–Altman plot between EQ-5D-5L and SF-6D

Table 2 shows the mean scores of the EQ-5D-3L, EQ-5D-5L, and SF-6D in the general population classified according to sex and age category. The QOL score measured using the SF-6D was significantly lower than those measured using the EQ-5D scores. The ICC was 0.802 between EQ-5D-3L and EQ-5D-5L, 0.249 between EQ-5D-3L and SF-6D, and 0.234 between EQ-5D-5L and SF-6D, respectively. The Bland–Altman plot between EQ-5D-5L and SF-6D is shown in Fig. 1. This plot indicates that outliers (SF-6D scores that are higher than the EQ-5D scores) exist for lower QOL scores.

**Table 2:** Summary statistics of QOL scores

Age (years)	Male			Female		
	EQ-5D-3L	EQ-5D-5L	SF-6D	EQ-5D-3L	EQ-5D-5L	SF-6D
20–29						
*N*	100	100	95	98	98	98
Mean	0.947	0.945	0.731	0.946	0.950	0.727
SD	0.114	0.102	0.136	0.112	0.084	0.133
30–39						
*N*	76	76	76	85	86	86
Mean	0.957	0.950	0.729	0.933	0.937	0.695
SD	0.098	0.080	0.125	0.121	0.089	0.114
40–49						
*N*	88	88	88	95	95	93
Mean	0.948	0.941	0.704	0.917	0.914	0.688
SD	0.129	0.088	0.124	0.134	0.102	0.128
50–59						
*N*	88	88	87	102	102	98
Mean	0.936	0.936	0.741	0.921	0.928	0.704
SD	0.121	0.101	0.135	0.130	0.092	0.129
60–69						
*N*	101	101	98	100	101	101
Mean	0.896	0.911	0.691	0.881	0.899	0.658
SD	0.156	0.158	0.141	0.144	0.105	0.112
≥70						
*N*	105	104	101	103	102	99
Mean	0.853	0.866	0.674	0.808	0.828	0.635
SD	0.164	0.155	0.137	0.202	0.202	0.129

In Table 3, the results of the ANOVA, including socio-demographic factors, are presented. The measured QOL scores of people older than 60 years of age were significantly lower than those of younger people when calculated using all three measures. The QOL scores of women tended to be slightly lower than those of men. Considering other socio-demographic factors, a lower household income (<2 million JPY) was associated with a lower QOL score, even after adjustments for sex and age. A shorter education period also influenced the QOL score, but the QOL scores did not differ among people who had received an education beyond high school. Marital status and employment pattern (full time, part time or self-employment) were not correlated with the QOL score.

**Table 3:** Relation between QOL scores and socio-demographic characteristics

	Model 1			Model 2		
	EQ-5D-3L	EQ-5D-5L	SF-6D	EQ-5D-3L	EQ-5D-5L	SF-6D
Intercept	0.9574	0.9551	0.7430	0.8516	0.8434	0.6535
Age (years)						
20–29	–	–	–	–	–	–
30–39	−0.0018	−0.0041	−0.0177	0.0019	−0.0024	−0.0142
40–49	−0.0146	−0.0201	−0.0336*	−0.0205	−0.0236	−0.0353*
50–59	−0.0182	−0.0150	−0.0076	−0.0234	−0.0186	−0.0115
60–69	−0.0581*	−0.0426*	−0.0552*	−0.0566*	−0.0432*	−0.0493*
≥70	−0.1160*	−0.1000*	−0.0752*	−0.0915*	−0.0743*	−0.0618*
Sex						
Male	–	–	–	–	–	–
Female	−0.0218*	−0.0156*	−0.0273*	−0.0211*	−0.0147	−0.0268*
Household income (JPY 10,000)						
<100				–	–	–
100–200				0.0060	0.0269	0.0071
200–400				0.0547*	0.0652*	0.0582*
400–600				0.0616*	0.0731*	0.053488*
600–1000				0.0577*	0.0774*	0.0603*
1000–1500				0.0613*	0.0716*	0.0586*
1500–2000				0.0904*	0.0942*	0.1106*
>2000				0.0934*	0.1095*	0.0747
Education						
Elementary or junior high				–	–	–
High school				0.0531*	0.0499*	0.0402*
College				0.0518*	0.0438*	0.0393*
University or graduate				0.0675*	0.0493*	0.0396*

* *P* value < 0.05

**Fig. 2:**
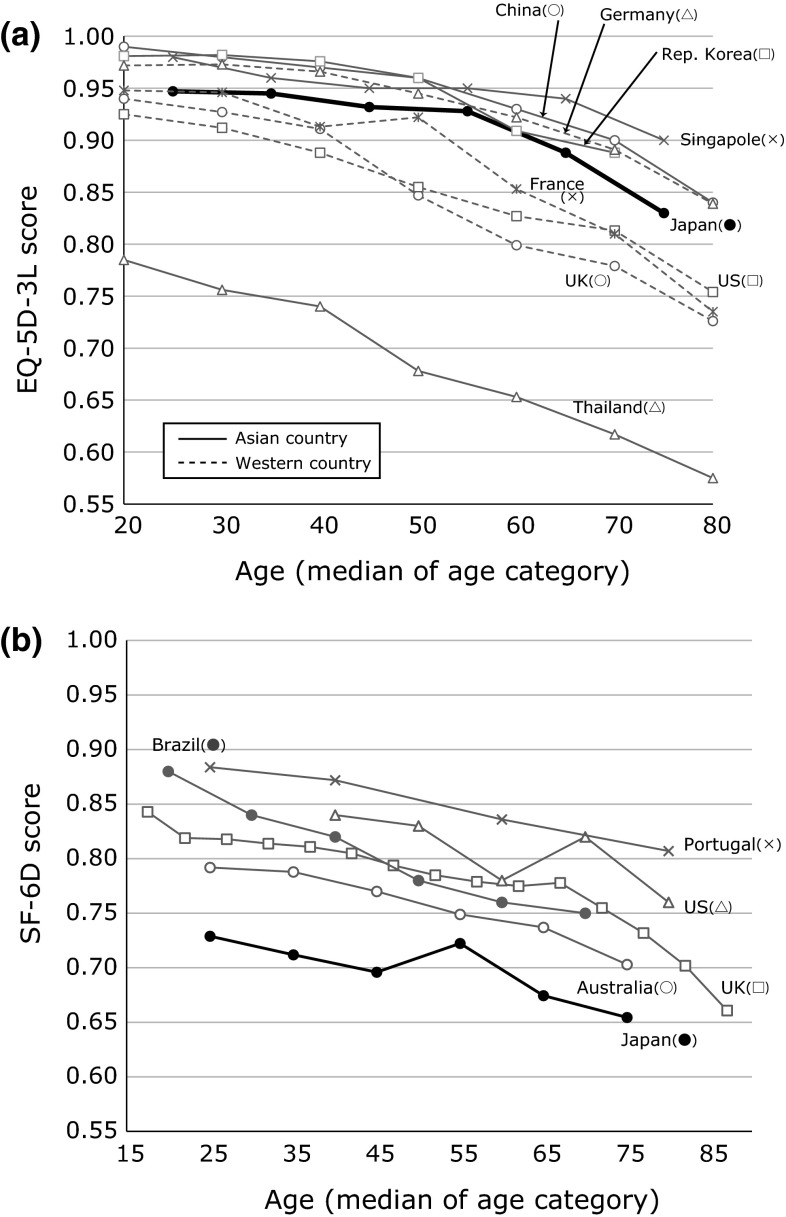
Comparison of Japanese population norms with those of other countries. **a** EQ-5D-3L, **b** SF-6D

A comparison with the population norms for the EQ-5D-3L and SF-6D in other countries is shown in Fig. 2. The figure shows the relation between the mean QOL score of both sexes and the median age category based on published reports [EQ-5D-3L (country-specific value set): Szende et al. [12] except Singapore [29], SF-6D: already shown in the Introduction section]. The Japanese population norms for the EQ-5D tended to be lower than those in some countries (China, Korea, Singapore, and Germany) and to be higher than others (USA, UK, France, and Thailand). On the other hand, the SF-6D score was the lowest among the other countries (USA, UK, Australia, Portugal, and Brazil) for which population norms are available.

### Percentage of respondents reporting full health

**Fig. 3:**
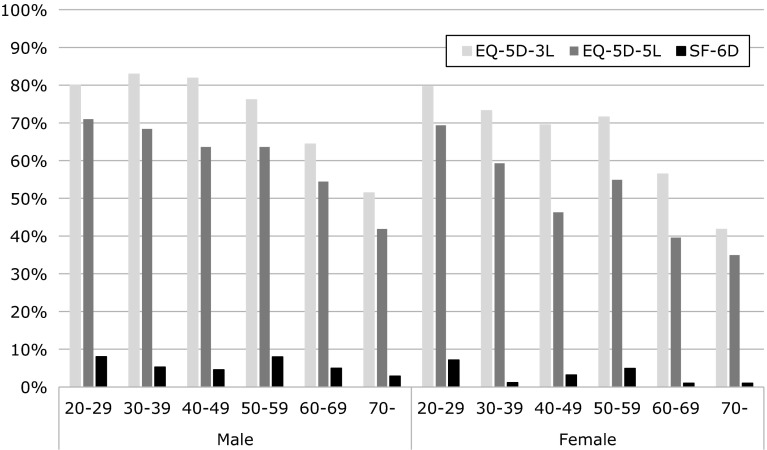
Percentage of respondents reporting full health

The percentages of respondents reporting full health were 68 % when measured using the EQ-5D-3L (80 % for subjects in their 20 s, 78 % in their 30 s, 75 % in their 40 s, 74 % in their 50 s, 60 % in their 60 s, and 47 % in their 70 s or older) and 55 % when measured using the EQ-5D-5L (70, 64, 55, 59, 47, and 38 % for the respective age categories); however, 4 % (8, 3, 4, 6, 3, and 2 % for the respective age categories) reported full health when measured using the SF-6D (Fig. 3). Table 4 shows the percentages of respondents with any problem in each dimension of the EQ-5D-3L, the EQ-5D-5L, and the SF-6D. Among younger people’s responses for the EQ-5D, the percentages of pain/discomfort and anxiety/depression were higher than those of other dimensions, which mainly correspond to physical and/or social function. When both sexes were compared, the percentage of women with any problem in the pain/discomfort dimension was significantly higher than that for men regardless of age. In addition, the EQ-5D-5L detected more health problems than the 3L in almost all the dimensions independently of the sex and age categories. Respondents chose a not-full state on the SF-6D more frequently than on the EQ-5D. For example, in almost all the sex and age categories, approximately 50–70 % of respondents reported a problem in the pain dimension, 60–80 % in the mental health dimension, and 80–90 % in the vital dimension.

**Table 4:** Percentage of respondents reporting any problem in each dimension

(a) EQ-5D				
Age (years)	Male		Female	
	3L (%)	5L (%)	3L (%)	5L (%)
20–29				
MO	3	2	4	3
SC	1	2	2	1
UA	3	2	3	2
PD	10	20	13	22
AD	14	22	11	19
30–39				
MO	1	3	2	3
SC	0	1	0	0
UA	1	1	3	3
PD	13	22	22	35
AD	9	24	10	17
40–49				
MO	2	2	5	7
SC	1	1	1	1
UA	6	7	8	8
PD	13	28	23	43
AD	8	20	16	27
50–59				
MO	6	6	6	8
SC	1	2	0	1
UA	8	9	5	6
PD	16	32	27	42
AD	13	18	11	16
60–69				
MO	10	12	11	13
SC	4	4	4	6
UA	11	13	13	15
PD	33	43	37	56
AD	10	13	18	23
≥70				
MO	24	34	32	37
SC	11	13	11	12
UA	27	25	26	27
PD	35	50	50	61
AD	15	19	12	20

(b) SF-6D		
Age (years)	Male (%)	Female (%)
20–29		
PF	26	32
RL	15	13
SF	28	22
BP	49	56
MH	74	76
VT	85	84
30–39		
PF	45	56
RL	11	19
SF	22	30
BP	55	64
MH	68	70
VT	88	94
40–49		
PF	56	58
RL	17	28
SF	30	34
BP	58	69
MH	70	80
VT	91	90
50–59		
PF	63	74
RL	23	20
SF	22	33
BP	55	67
MH	62	64
VT	85	88
60–69		
PF	76	92
RL	32	43
SF	39	46
BP	73	84
MH	59	66
VT	86	87
≥70		
PF	91	96
RL	39	48
SF	31	43
BP	71	81
MH	57	57
VT	85	85

*MO* mobility, *SC* self-care, *UA* usual activities, *PD* pain/discomfort, *AD* anxiety/depression, *PF* physical functioning, *RL* role limitation, *SF* social functioning, *BP* bodily pain, *MH* mental health, *VT* vitality

### Influence of diseases and symptoms on QOL score

Table 5 shows the relations between the QOL scores and both the diseases and symptoms that the respondents felt were the most important to them. Among the diseases, “depression or mental diseases,” “stroke,” and “rheumatoid arthritis” had the largest influence on the QOL score. These diseases decreased the QOL by 0.15–0.2. On the other hand, “dyslipidemia,” “hypertension,” and “tooth disorder” had a minimal impact on the QOL score, although their prevalence was relatively high. Considering the prevalence of diseases (decrease in the QOL score multiplied by the number of respondents), “depression or mental diseases,” “lumbago,” and “diabetes” were the top three diseases, decreasing the QOL score at the general population level. The QOL scores of respondents with some symptoms, such as “sleeplessness,” “arthritic pain,” and “having trouble moving limbs,” were lower than those of respondents reporting other symptoms.

**Table 5:** Relation between QOL scores and diseases and symptoms

		EQ-5D-3L	EQ-5D-5L	SF-6D
(*a*) *Diseases*				
Intercept		0.9631	0.9618	0.7572
Age (years)				
20–29	192	–	–	–
30–39	160	0.0134	0.0046	−0.0077
40–49	189	−0.0034	−0.0079	−0.0257
50–59	185	0.003	−0.0016	0.0061
60–69	202	−0.0232	−0.0167	−0.0296*
≥70	214	−0.0552*	−0.0384*	−0.0308*
Sex				
Male	556	–	–	–
Female	586	−0.0185*	−0.0102	−0.0229*
Diseases				
No diseases	608	–	–	–
Diabetes	42	−0.0893*	−0.0898*	−0.0558*
Dyslipidemia	24	−0.0045	−0.0192	−0.0353
Depression or mental diseases	22	−0.1934*	−0.1739*	−0.1845*
Eye diseases	16	−0.0677*	−0.0543*	−0.0420
Ear disorder	8	−0.0390	−0.0293	−0.1088*
Hypertension	94	−0.0236	−0.0181	−0.0402*
Stroke	8	−0.2361*	−0.1675*	−0.1370*
Angina or myocardial infarction	18	−0.0707*	−0.0642*	−0.1054*
Cold	7	−0.0343	−0.0356	−0.1264*
Stomach and duodenal diseases	9	−0.0878	−0.0823*	−0.0941*
Tooth disorder	45	−0.0207	−0.0259	−0.0494*
Rheumatoid arthritis	9	−0.1786*	−0.1835*	−0.0846*
Arthrosis	15	−0.1472*	−0.1173*	−0.0929*
Omalgia	19	−0.0260	−0.0659*	−0.0856*
Lumbago	37	−0.1114*	−0.1026*	−0.0963*

	*n*	EQ-5D-3L	EQ-5D-5L	SF-6D
(*b*) *Symptoms*				
Intercept		0.9912	0.9844	0.7893
Age (years)				
20-29	192	–	–	–
30–39	160	0.0020	−0.0013	−0.0172
40–49	189	−0.0104	−0.0167	−0.0326*
50–59	185	−0.0111	−0.0157	−0.0055
60–69	202	−0.0409*	−0.0292*	−0.0378*
≥70	214	−0.0800*	−0.0664*	−0.0510*
Sex				
Male	556	–	–	–
Female	586	−0.0173*	−0.0085	−0.0216*
Symptoms				
No symptoms	588	–	–	–
Having a fever	6	−0.1211*	−0.0890*	−0.0885
Feeling sluggish	8	−0.1481*	−0.1382*	−0.2050*
Sleeplessness	12	−0.2012*	−0.1637*	−0.2078*
Feeling irritable	14	−0.0661	−0.0915*	−0.1503*
Headache	25	−0.1205*	−0.1102*	−0.1130*
Dizziness	11	−0.1152*	−0.1125*	−0.1569*
Blurred vision	12	0.0220	−0.0019	−0.0738*
Having trouble seeing	13	−0.0778*	−0.0512	−0.1457*
Buzzing	9	−0.0501	−0.0719*	−0.0663
Having trouble hearing	9	−0.1331*	−0.0641	−0.1362*
Palpitation	10	−0.1300*	−0.1022*	−0.1430*
Cough and/or sputum	24	−0.0395	−0.0264	−0.0919*
Nasal congestion or mucus	43	−0.0509*	−0.0296	−0.0638*
Feeling indigestion or heartburn	10	−0.0863*	−0.0828*	−0.0870*
Abdominal or stomach ache	12	−0.0846*	−0.0844*	−0.1176*
Dental pain	10	−0.0436	−0.0113	−0.0526
Itching (such as eczema, tinea pedis)	13	−0.0543	−0.0985*	−0.0534
Stiff shoulders	67	−0.0482*	−0.0478*	−0.1007*
Back pain	82	−0.1142*	−0.1049*	−0.1303*
Arthritic pain	40	−0.1831*	−0.1247*	−0.1274*
Having trouble moving limbs	10	−0.2747*	−0.3618*	−0.2008*
Numbness of limbs	15	−0.1153*	−0.1092*	−0.1255*
Coldness of limbs	11	−0.1645*	−0.1566*	−0.1389*
Frequent urination	10	−0.0876*	−0.0846*	−0.1183*

* *P* value < 0.05

The differences in the QOL scores between respondents with and those without any diseases were 0.064 for measurements based on the EQ-5D-3L, 0.061 for measurements based on the EQ-5D-5L, and 0.073 for measurements based on the SF-6D, which is regarded as the between-group MID in the general population. If symptoms were used in the same analysis, the differences were 0.093 for both the EQ-5D-3L and EQ-5D-5L and 0.112 for the SF-6D. Considering our results, the between-group MID can be estimated to range between 0.05 and 0.1 for all three measures.

## Discussion

To our knowledge, this is the first study to examine the Japanese population norms of three preference-based QOL measures: the EQ-5D-3L, EQ-5D-5L, and SF-6D. Sampling was based on the Basic Resident Register data for each municipality. This sampling is regarded as one of the most rigid and reliable methods in Japan. The reason for the differences in the QOL scores, compared with the population norms in other countries, is unclear; however, the differences may be influenced by (a) differences in actual health states, (b) differences in the value sets used in each country, and/or (c) differences in the degree of the ceiling effect or other characteristics. The ceiling effect of the EQ-5D-3L (especially for pain/discomfort among younger respondents) may be higher in the present study than in western countries [12]. Of note, the difference in the population norms does not necessarily indicate a difference in the respondents’ health states.

The results are shown stratified according to sex and age category. The QOL scores were significantly reduced if the respondents were older than 60 years of age, female, had a lower income, or a shorter period of education. According to our results, a larger income was associated with a higher QOL score. The causal relation (whether poverty causes a poor health state or a poor health state is the cause of poverty) is unclear, but this finding may be useful for public health policies. This relation was observed in other countries. For example, in the USA [14], the QOL score as measured using the EQ-5D-3L was 0.81 for the poorest category (≤USD 10,000), although it was 0.92 for the richest (≥USD 75,000).

The percentage of reports of any health problem for the EQ-5D-5L is higher than that for EQ-5D-3L in almost all the sex and age categories. Some authors have pointed out that the EQ-5D-3L has a ceiling effect, which is defined as “the proportion of respondents scoring ‘no problems’ on any of the five dimensions” [45], because the instrument lacks enough sensitivity. A three-level questionnaire allows respondents with a slightly worsened health state to be reported as having a full health state. This is one example of how the ceiling effect problem has been improved by the revision of the EQ-5D-3L, resulting in the EQ-5D-5L. According to Table 2, the standard deviation of the QOL score measured using the EQ-5D-5L tended to be smaller than that measured using the EQ-5D-3L. This result may also arise from the increased number of levels, enabling respondents to choose intermediate levels.

Compared with the EQ-5D measures, the QOL score measured using the SF-6D was lower in the general population. A poor agreement between the EQ-5D and the SF-6D scores was observed, with a low ICC of 0.249 (EQ-5D-3L) and 0.234 (EQ-5D-5L). One cause seems to be clear, considering the percentages of respondents with full health as shown in Table 4. The percentage of people who chose no problem on the SF-6D was much lower than that for either EQ-5D measure. This result may be characteristic of the SF-6D and not only for the Japanese population. In Australia [33], the proportions of respondents in the 18- to 30-year age category who reported any problem in each dimension were as follows: 32 % for PF, 23 % for RL, 39 % for SF, 60 % for BP, 49 % for MH, and 94 % for VT. On the other hand, a Bland–Altman plot indicated that most outliers (an SF-6D score that was higher than the EQ-5D score) occurred at lower QOL scores. These tendencies were similar to those reported by Kontodimopoulos et al. [46] in Greece. Thus, the SF-6D may have a floor effect [47], i.e., the lowest QOL score of the SF-6D (0.292) is higher than that of the EQ-5D-5 (−0.025).

The Japanese population norms for the SF-6D seem to be lower than those for other countries, although that of EQ-5D-3L is similar to those of other countries (except Thailand). It is unclear whether this lower score is a result of the Japanese response pattern or a Japanese tariff for the SF-6D. According to these results, if the QOL score is used for economic evaluations, its interchangeability should be carefully considered [48–52], since the baseline scores of the general population differ between the Japanese EQ-5D and the SF-6D.

We analyzed the differences in the QOL scores between respondents with diseases/symptoms and those without diseases/symptoms by comparing the cross-sectional between-group MID of each measure. The anchor-based MID is more commonly measured longitudinally across multiple time points, which is closer to the definition of MID. In the general population, repeated surveys are more difficult to perform than in clinical trials. Of note, our estimated score may not be the same as the intra-respondent MID. However, the between-group MID may be more useful when the results of between-group differences have been interpreted. Walters et al. [53] showed that the mean MID of the SF-6D was 0.041 and that of the EQ-5D-3L was 0.074 in a review of studies. In cancer patients, the MID of the EQ-5D was estimated to be 0.08 (UK score) and 0.06 (US score), and these values were anchored to the performance status and the FACT-G score [54]. According to a study examining post-traumatic stress disorder (PTSD), the MID was calculated as 0.05–0.08 (anchor-based method) and 0.04 to 0.10 (distribution method) [55]. Considering these scores, our MID is consistent with previous studies.

A limitation of this study was its relatively small sample size, compared with other studies to identify population norms. We think that the sample number was sufficient to estimate the population norms according to sex and age category, considering the interpretable and consistent results with previous studies in other countries. However, a larger number of subjects may enable a clearer relation between the QOL score and diseases/symptoms to be identified. Furthermore, analyses of the effects of diseases with small prevalence could not be performed. Another limitation is the order in which the three instruments were presented to the respondents. As the order was fixed, and not randomized, the possible influence of the order on the results cannot be excluded based only on our data.

In conclusion, we demonstrated the following characteristics of three preference-based measures: (a) the Japanese population norms according to sex and age category, (b) the relation between QOL scores and socio-demographic factors, (c) the reliability of the three measures in the general Japanese population, (d) the percentage of reports of any problem, (e) the influence of diseases/symptoms on the QOL scores, and (f) the between-group MID. The respondents were randomly collected from all eight regions of Japan in a door-to-door survey, and the representativeness of the sample was considered to be good. The resulting information may be useful for calculating QALY in economic evaluations and research examining QOL score.
